# Extracardiac vagal nerve stimulation–guided cardioneuroablation in patients with interrupted superior vena cava and brachiocephalic vein drainage: Tips, tricks, and insights from 500 consecutive cases including 5 complex procedures—Insights from the Polish Cardioneuroablation and Cardioneuromodulation registry

**DOI:** 10.1016/j.hrcr.2026.01.010

**Published:** 2026-01-27

**Authors:** Sebastian M. Stec, Antoni Wileczek, Hasso Uuetoa, Marta Kornaszewska, Magdalena Zagrodzka, Jakub Batko, Karolina Barańska-Pawełczak

**Affiliations:** 1 Department of Cardiac Surgery and Transplantology, National Medical Institute of the Ministry of Interior and Administration, Warsaw, Poland; 2 ELMedica EP-Network, Skarżysko-Kamienna, Poland; 3 CardioNeuroLab, Sabamed Medical Center, Krasne, Rzeszow, Poland; 4 Department of Cardiology, Sahlgrenska University Hospital, Gothenburg, Sweden; 5 Tallin, Private Cardiac Outpatient Clinic, Estonia; 6 Cardiovascular Medical Centre, Kleczany/Nowy Sącz, Poland; 7 Department of Diagnostic Imaging – Quadia, Piaseczno, Poland; 8 Department of Anatomy, Jagiellonian University Medical College, Krakow, Poland; 9 Department of Cardiology, Specialistic Hospital in Zabrze, Zabrze, Poland

**Keywords:** Cardioneuroablation, Extracardiac vagal nerve stimulation, Superior vena cava obstruction, Pacemaker lead–related venous obstruction, Superior vena cava syndrome, Vagally mediated bradyarrhythmias, Reflex syncope

**Key Teaching Points**

• Patients with vagally mediated bradyarrhythmias (VMBs) referred for extracardiac vagal nerve stimulation (ECVS)–guided cardioneuroablation (CNA) typically require retrograde and bilateral cannulation of the internal jugular veins (IJVs) via the superior vena cava (SVC). However, rare anomalies in SVC and brachiocephalic vein drainage can hinder the implementation of ECVS for CNA guidance, necessitating alternative vascular access for ECVS, preferably bilateral but at minimum unilateral.
• Preprocedural multimodality imaging (computed tomography, magnetic resonance imaging, transesophageal echocardiography), complemented by targeted venography, is crucial for identifying SVC and brachiocephalic venous interruptions enabling individualized ECVS-guided CNA strategies and coordinated Heart Team planning.
• This case series describes the incidence of these anomalies, explores alternative vascular access routes, and provides technical tips, procedural strategies, and imaging guidance for successful IJV cannulation in patients with VMB and interrupted SVC drainage into the right atrium (RA) referred for complex ECVS-guided CNA. Access via unilateral or bilateral subclavian veins, a persistent left SVC, the azygos vein, or retrograde puncture with ultrasonography-guided IJV cannulation can facilitate ECVS-guided CNA in patients with complex upper thoracic venous anatomy owing to adult congenital heart disease or iatrogenic interruption of SVC–RA drainage.

## Introduction

Extracardiac vagal nerve stimulation (ECVS) is a novel technique used to assess the impact of vagal reflexes on sinoatrial and atrioventricular node automaticity and conduction. This typically manifests as sinus asystole, severe or prolonged sinus bradycardia, and advanced or complete atrioventricular block (AVB). ECVS is also used in the management of carotid sinus syndrome with a cardioinhibitory reflex or selected cases of persistent atrial fibrillation (AF).^1^, ^2^, ^3^ Patients with vagally mediated bradyarrhythmias (VMBs) referred for ECVS-guided cardioneuroablation (CNA) usually require retrograde, bilateral cannulation of the internal jugular vein (IJV), typically via the right atrium (RA) and superior vena cava (SVC). During ECVS-guided CNA, baseline, periprocedural, and postprocedural ECVS may be used to evaluate the selective effects of CNA on the sinoatrial or atrioventricular node. Complete binodal and bilateral parasympathetic denervation is confirmed by the absence of ECVS-induced reflexes (sinus arrest and AVB) from the left and right IJVs.^4^

However, interruption of SVC drainage into the RA and further interruption between the brachiocephalic vein and the SVC pose significant challenges to performing ECVS and necessitate alternative vascular access—preferably bilateral, but at minimum unilateral. Several techniques can enhance the success of IJV cannulation, including the use of vascular guidewires, stabilizing or steerable transseptal sheaths, multipurpose vascular guiding sheaths, and physiological maneuvers such as positive end-expiratory pressure to extend upper venous structures. Despite these methods, ultrasound-guided ECVS still requires visualization of the intravascular catheter along the cervical course of the IJV and confirmation of its close proximity to the vagus nerve. Although left-sided ECVS is performed significantly less frequently, the reason for this limitation remains unclear, and no additional techniques have been reported to improve its success rate.

This case series presents alternative approaches to IJV cannulation, based on the first 500 consecutive ECVS-guided CNA procedures performed between 2018 and 2025, either directly by the first operator (S.M.S.) or under his supervision as a proctor for physicians from Poland, Sweden, Latvia, Estonia, Turkey, Serbia, and Germany. This series aimed to introduce and demonstrate the use of various imaging modalities and alternative vascular access strategies for performing ECVS-guided CNA in patients with VMB, despite anatomic anomalies of SVC and brachiocephalic vein drainage.

## Baseline characteristics of ECVS implementation in patients with VMB referred for CNA

Between June 2018 and August 2025, standard baseline unilateral ECVS via the SVC and brachiocephalic vein was successfully performed in at least 1 IJV (right sided in 99.0% of cases [490 of 495]) in the first 500 patients with VMB referred for ECVS-guided CNA. All patients had electrocardiographically (ECG) documented bradyarrhythmias confirmed by tilt-table testing, prolonged ECG monitoring, or an implantable loop recorder, along with a positive response to atropine, defined as either a ≥30% increase in sinus rhythm or significant improvement in atrioventricular conduction, including resolution of reproducible second-degree or advanced AVB. The standard CNA protocol included a baseline electrophysiological study (EPS) and evaluation of a His bundle electrogram during incremental atrial pacing. In all patients, particularly those considered at high risk (eg, syncope with injury, syncope while driving, high-risk occupational status, or candidates for transvenous lead extraction [TLE] after CNA), bilateral postprocedural ECVS was attempted at the end of the procedure. In addition, a second EPS and ECVS were recommended before TLE and occupational medicine clearance for return to work in high-risk professions. In 5 of the 500 patients (1.0%), rare anomalies of SVC and brachiocephalic vein drainage impaired the standard implementation of baseline or postprocedural bilateral ECVS. Therefore, either ad hoc or prespecified alternative vascular access routes for unilateral or bilateral ECVS were implemented when standard access was not feasible. All patients provided a written informed consent, including permission for the use of their imaging data in publications. Patients treated in Poland were enrolled in the Polish Cardioneuroablation and Cardioneuromodulation registry. Patients treated in Poland were enrolled in the Polish Cardioneuroablation and Cardioneuromodulation registry (NCT07196397) approved by the Bioethics Committee of the Regional Medical Chamber in Rzeszów, Poland (51/20/B24). The study complies with the Declaration of Helsinki.

## Case 1 (congenital anomaly): Persistent left SVC drainage into the coronary sinus with a coexisting right SVC

A 51-year-old fit and otherwise healthy man was referred for cardiovascular autonomic assessment owing to 2 recent episodes of severe vasovagal syncope (VVS) without prodromal symptoms, along with frequent presyncope (∼1 episode per month) and daily palpitations. A 12-lead resting ECG and 24-hour Holter monitoring revealed normal sinus rhythm (minimum 44 beats/min [bpm]; average 70 bpm; maximum 125 bpm), accompanied by symptomatic frequent premature ventricular contractions (PVCs), PVC couplets, and nonsustained ventricular tachycardia (VT) with a typical right ventricular outflow tract morphology (2500 PVCs/24 hours). Transthoracic echocardiography (TTE), performed by a general cardiologist, showed no significant structural abnormalities. The patient subsequently underwent comprehensive cardiovascular autonomic testing (CAT), culminating in a head-up tilt test, which revealed 17 seconds of sinus asystole. This confirmed a diagnosis of VVS with a cardioinhibitory reflex and prolonged asystole, effectively excluding frequent PVCs or nonsustained VT as the primary cause of syncope. The atropine test was positive, with a normal sinus rate increasing from 50 to 80 bpm within 10 minutes after an intravenous bolus of atropine (0.02 mg/kg). Left subclavian vein access for coronary sinus (CS) cannulation and decapolar catheter placement revealed a persistent left SVC (PLSVC) draining directly into the CS, with no connection between the left and right brachiocephalic veins. Cannulation of the right femoral vein, performed without deep sedation, triggered an additional 4–6 seconds of sinus asystole. Despite this, successful access to the right IJV via the persistent right SVC was achieved using a guidewire and a long sheath, enabling ECVS.

After successful ablation of PVCs and nonsustained VT originating from the right ventricular outflow tract, ablation of 6 presumed ganglionated plexi (GP) areas was performed. Subsequently, the ablation catheter was advanced into the left IJV via retrograde cannulation of the PLSVC and the distal CS. 4 left-sided ECVS attempts were conducted: 2 at the level of the jugular foramen under fluoroscopic guidance and 2 along the cervical course of the IJV. No ECG response was observed, and each left-sided ECVS attempt was associated with the induction of a tearing sensation reported by the patient. Postprocedural TTE, with detailed CS assessment, revealed an enlarged CS and primary flow of agitated saline from the left ulnar vein into the CS and subsequently into the RA. Cardiac magnetic resonance imaging confirmed the periprocedural findings, demonstrating direct drainage of the PLSVC into the CS. At 24-month follow-up, the patient remained asymptomatic, and multiple 24-hour ECG Holter recordings showed complete resolution of PVCs, PVC couplets, and nonsustained VT.

## Case 2 (congenital anomaly): PLSVC drainage into the CS

**Figure 1:**
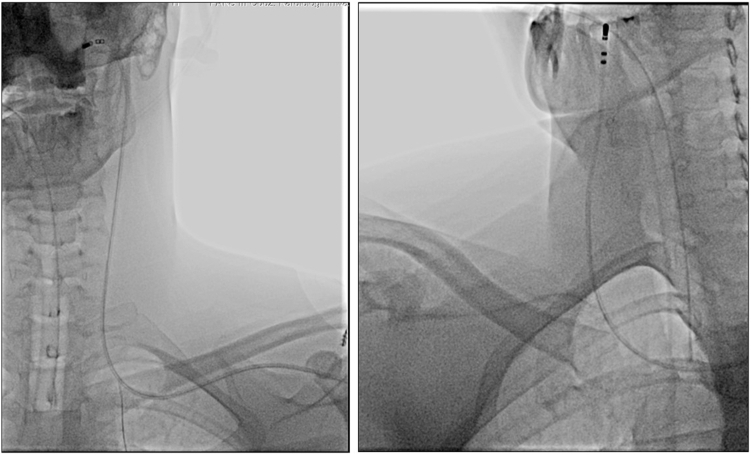
Left and right extracardiac vagal nerve stimulation performed via left subclavian vein access.

A 31-year-old otherwise healthy man was referred for complex CNA owing to symptomatic sinus bradycardia (manifesting as presyncope, tiredness, and fatigue), which limited the use of antiarrhythmic drugs. He also had a 5-year history of symptomatic paroxysmal AF, characterized by palpitations, dyspnea, and exercise intolerance (European Heart Rhythm Association class 2b/3). Preprocedural TTE revealed an enlarged CS, raising suspicion of PLSVC drainage. This finding was confirmed by computed tomography, which clearly visualized PLSVC draining into the CS. The patient was scheduled for elective CNA under transesophageal echocardiographic (TEE) guidance. Using a right femoral vein approach, a mapping catheter was advanced through the CS and PLSVC into the left brachiocephalic and left subclavian veins. Attempts to cannulate the left and right IJVs were initially unsuccessful; however, access via the left subclavian vein allowed retrograde cannulation of both IJVs (Figure 1). A reproducible effective refractory period of 400 + 180 ms was confirmed using the CS catheter. A vagal AF induction test was performed, which did not induce AF but resulted in the shortening of the effective refractory period to 400 + 150 ms. Owing to the inability to use standard SVC maneuvers with a long transseptal sheath from the SVC to the fossa ovale, transseptal puncture was performed under TEE guidance. 2 transseptal sheaths were successfully advanced into the left atrium. CNA was completed by targeting 6 typical GP regions, along with pulmonary vein isolation of all 4 veins. Subsequently, fluoroscopy- and ultrasound-guided ECVS attempts from the left subclavian vein confirmed the disappearance of vagal reflexes, indicating a successful procedural outcome. During 12 months of follow-up with smartphone-based ECG monitoring, the patient remained free of AF recurrence and symptomatic bradycardia.

## Case 3: Lead-related occlusion at the left brachiocephalic–SVC junction

A 36-year-old man with a history of symptomatic sinus bradycardia and recurrent syncope was eventually diagnosed as having sinus asystole and vasovagal syncope, confirmed by an implantable loop recorder. Noninvasive, nonpharmacologic, and pharmacologic therapies were ineffective, and a permanent dual-chamber pacemaker (DDDR) was implanted via the left subclavian vein in 2022. 3 years later, the patient sought an alternative to permanent pacing owing to professional restrictions related to pacemaker therapy. An atropine challenge revealed a marked increase in baseline sinus rate from 45 to 85 bpm, with a normal PQ interval. The patient was referred for CNA. Using right femoral vein access, baseline ECVS was performed via right IJV cannulation. Stimulation elicited 6 seconds of sinus asystole, 5 seconds of complete AVB, and an additional 5 seconds of 2:1 AVB. CNA was performed in 6 standard GP sites, resulting in an increase of sinus rate from 50 to 80 bpm and complete elimination of vagal reflexes during repeat right-sided ECVS. Left-sided ECVS was abandoned after several unsuccessful attempts to cross a lead-related obstruction between the left brachiocephalic vein and the SVC. During a subsequent elective procedure, direct retrograde access to the left IJV was achieved, allowing for detailed venography of the left upper thoracic venous system. Control EPS and bilateral ECVS demonstrated sinus node denervation but persistent vagal response at the atrioventricular node, with transient 2:1 or 3:1 AVB during left-sided ECVS (proximal CS pacing at 600 ms). Targeted left-sided ablation of the left superior pulmonary vein GP and left inferior para-Hisian GP resulted in complete bilateral atrioventricular node denervation, as confirmed by ECVS. The patient subsequently underwent CAT and noninvasive EPS with atropine challenge, both of which showed no abnormalities. After shared decision making, he was referred for elective TLE.

## Case 4: Severe SVC syndrome owing to lead-related SVC stenosis and left brachiocephalic vein occlusion

A 47-year-old woman with a DDDR pacemaker implanted over 7 years earlier for recurrent syncope with associated head trauma was referred for evaluation. A tilt-table test revealed a mixed-type vasovagal response at 14 minutes as the primary mechanism of syncope, characterized by sinus bradycardia, hypotension, and a 32-second asystole. After DDDR implantation, the patient’s clinical course improved significantly, and she remained asymptomatic for several years. However, since late 2023, she developed symptoms consistent with SVC syndrome (SVCS), including swelling and redness of the head and neck while lying down—especially in the prone position or when leaning forward, accompanied by congestion-related headaches and a sensation of facial and neck pressure. Venography of the upper chest revealed an occlusion of the left brachiocephalic vein and a short segment of stenosis in the SVC just below the confluence of the brachiocephalic veins. After a shared decision-making process, CNA was proposed as the initial intervention, followed by TLE and dilatation of the brachiocephalic vein and the SVC. Discontinuation of permanent pacemaker therapy was planned if CNA effectively resolved symptoms of VVS and other bradycardias. If necessary, implantation of a leadless AAIR or DDDR system was to be considered. An intravenous atropine test (0.02 mg/kg) increased the sinus rate from 50 (AAI-paced rhythm) to 88 bpm (sinus rhythm) within 10 minutes. The test was considered positive, and the patient was referred for CNA.

CNA was performed under general anesthesia using right femoral vein access with 3 introducers. Initial attempts to advance a hydrophilic guidewire and CS catheter through the SVC were unsuccessful. Therefore, a retrograde approach to the right IJV, from caudal to cranial direction, was used. All femoral groin and cervical punctures were performed using a micropuncture set. At baseline, right-sided ECVS was performed in typical subcranial and cervical positions under ultrasound guidance. This induced prolonged sinus arrest and AVB (during proximal CS pacing). Left-sided access was abandoned owing to the risk of vascular injury. Subsequently, 3-dimensional electroanatomic mapping of the RA and left atrium was performed. Biatrial anatomic CNA targeting 6 GP regions was completed, with particular focus on septal GP sites, including the superior and inferior paraseptal GPs. Although an initial increase in sinus rhythm of more than 50% was observed (from 40 to 60 bpm), a subsequent gradual decline to 55 bpm occurred, leading to the decision to withhold further ablation applications. In addition, atrioventricular conduction improved, as evidenced by a shortening of the Wenckebach point from 460 to 400 ms, and corrected sinus node recovery time normalized from 662 to 469 ms.

These modest improvements were partially attributed to the effects of general anesthesia, as well as acute phenomena such as simultaneous parasympathetic and sympathetic denervation, or sinoatrial node stunning or injury observed immediately after ablation. Control right-sided ECVS demonstrated complete denervation, with no induction of sinus bradycardia, asystole, or AVB. During the postprocedural period, the patient required temporary atrial pacing support (AAI mode at 70 bpm). By the next day, her intrinsic heart rate stabilized at approximately 66 bpm, and the pacemaker was reprogrammed to DDD-ADI mode at 50 bpm. At 3 weeks after the procedure, her mean sinus rate was 75 bpm. However, a stress test (bicycle ergometry) revealed mild chronotropic incompetence, with a peak heart rate of only 122 bpm—approximately 70% of the age-predicted maximum. Despite this, the pacemaker had not delivered atrial or ventricular pacing during that period, with device interrogation showing an average sensed heart rate of 70 bpm. The pacemaker was subsequently reprogrammed to VVI mode at 30 bpm. Owing to mildly symptomatic chronotropic incompetence, the device was reprogrammed to AAIR mode (35–150 bpm) 6 weeks after the CNA procedure. 2 weeks later, remote device interrogation and teleconsultation revealed an atrial pacing burden of 7%, and the patient reported improved exercise tolerance. However, symptoms of SVCS worsened, and the patient began sleeping with 2 pillows to alleviate overnight headaches and facial congestion.

5 months after CNA, the patient underwent a repeat exercise stress test, which demonstrated improved functional capacity. Sinus rate increased from 74 to 144 bpm at peak exercise, corresponding to 83% of age-predicted maximum heart rate, indicating improved chronotropic response. At 6 months after CNA, TLE was successfully performed in a hybrid operating room. The procedure included simultaneous balloon angioplasty of the stenosed brachiocephalic vein and the SVC. Symptoms of SVCS resolved immediately after the procedure, and the patient reported further improvements in exercise capacity. Continued follow-up is planned at 3- to 6-month intervals.

## Case 5: Lead-related membranous obstruction at the SVC–RA junction with loss of brachiocephalic vein continuity in subclinical SVCS

A 34-year-old woman was referred for CNA. Her medical history included implantation of a dual-chamber permanent pacemaker (DDDR) via the left subclavian area for severe sinus bradycardia and recurrent syncope, with documented episodes of sinus asystole and syncope during 24-hour Holter monitoring. 1 month after pacemaker implantation, the patient experienced cardiogenic shock owing to atrial lead–related cardiac tamponade after physical activity. She underwent emergent atrial lead explantation and replacement of both the atrial lead and the generator. Her subsequent 9-year follow-up after DDDR system replacement was uneventful. Thereafter, the patient requested evaluation for elective discontinuation of permanent pacemaker therapy. At 3-month follow-up with the pacemaker programmed to VVI mode at 40 bpm, the patient demonstrated a ventricular pacing burden of less than 1%, with no episodes of syncope, although some prodromal episodes were reported. She subsequently underwent a comprehensive evaluation for VMB, including a noninvasive EPS, which was unremarkable, and a CAT.

**Figure 2:**
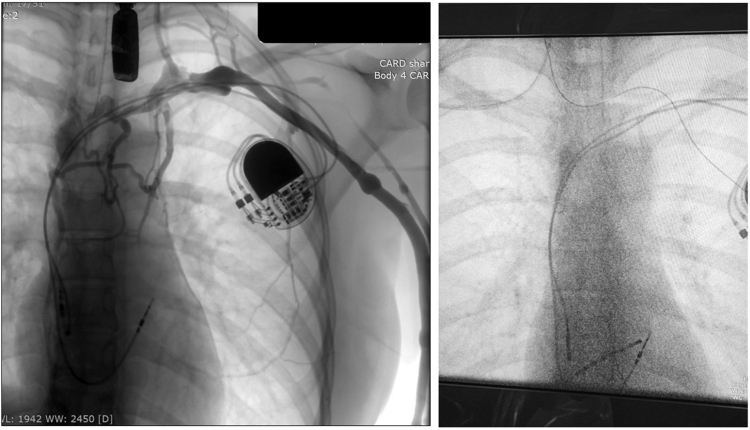
Case 5: venography and right internal jugular vein cannulation from right subclavian vein access.

During reprogramming to OVO mode, a head-up tilt test provoked a syncopal episode and revealed 8 seconds of sinus asystole, followed by prolonged sinus bradycardia (<10 bpm) lasting an additional 20 seconds. An atropine challenge demonstrated a significant chronotropic response, with an increase in sinus rate from 60 to 100 bpm and a normal PQ interval. The patient was subsequently referred for CNA. Using right femoral vein access, RA mapping revealed an interruption between the high RA and the SVC, which prevented the advancement of a long sheath, guidewire, ablation catheter, and nonsteerable decapolar catheter. Access via the right subclavian vein confirmed a complete lack of continuity between the SVC and RA, as well as the absence of communication between the left and right brachiocephalic veins. Venography demonstrated dominant azygos vein drainage into the inferior vena cava, consistent with iatrogenic, lead-related occlusion of the brachiocephalic vein (Figure 2). Retrograde cannulation of the right IJV was the only feasible option. Baseline ECVS performed via this access successfully induced sinus arrest and AVB.

CNA was performed except for the right septal region of the RA, including the presumed location of the superior paraseptal GP, owing to complete anatomic obstruction in that area. Subsequent detailed venography confirmed these anatomic findings and supported the diagnosis of subclinical SVCS. At 6-week follow-up, the patient remained asymptomatic. Control EPS, head-up tilt test, and atropine challenge were all negative. In addition, pacemaker interrogation during VVI mode at 30 bpm showed 0% ventricular pacing, confirming the short-term efficacy of the CNA. The patient was then referred for follow-up right-sided ECVS, EPS, and high-frequency stimulations of the left atrium. These evaluations revealed no abnormalities and no induction of asystole. Vascular access for right-sided ECVS was achieved not via the subclavian vein but through direct ultrasound-guided retrograde cannulation of the right IJV using a standard guidewire. Cannulation of the left IJV with the same approach was intentionally avoided to preserve access for potential future interventions. The patient subsequently underwent TLE under TEE guidance. During this complex hybrid procedure, balloon angioplasty of the membranous obstruction at the SVC–RA junction was performed, along with stenting of the left brachiocephalic vein. These interventions resulted in the restoration of flow through the high SVC–RA junction via a small residual opening in the iatrogenic membranous stenosis, and disappearance of compensatory azygos vein drainage. At 4-month follow-up, the patient remained asymptomatic with complete resolution of subclinical signs of SVCS, including facial and upper limb swelling and facial erythema triggered by bendopnea.

## Discussion

**Figure 3:**
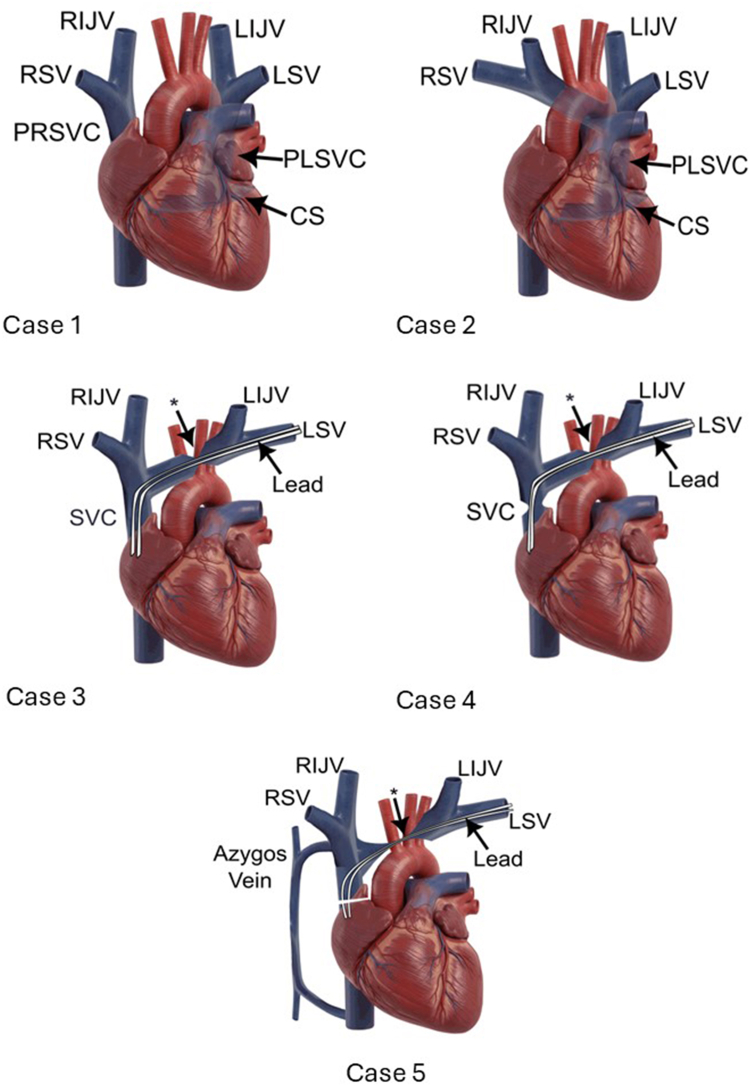
Anatomic diagrams of the presented cases. ∗Place of obstruction. CS = coronary sinus; LIJV = left internal jugular vein; LSV = left subclavian vein; PLSVC = persistent left superior vena cava; PRSVC = persistent right superior vena cava; RIJV = right internal jugular vein; RSV = right subclavian vein; SVC = superior vena cava.

**Figure 4:**
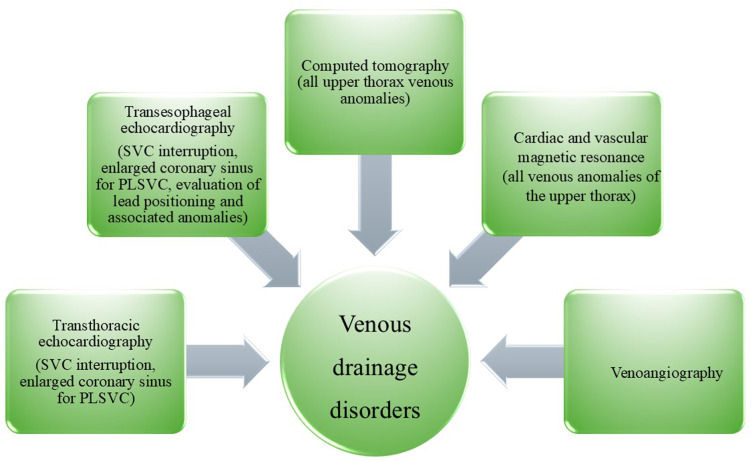
Recommended imaging modalities for diagnosing superior vena cava and/or brachiocephalic vein interruption in patients referred for extracardiac vagal nerve stimulation–guided cardioneuroablation. PLSVC = persistent left superior vena cava; SVC = superior vena cava.

Our case series demonstrates that alternative vascular access routes for IJV cannulation—enabling bilateral or unilateral ECVS (particularly left sided)—can be successfully achieved during complex CNA procedures in patients with interruption of the SVC and/or brachiocephalic veins (Figure 3). We also highlight the importance of using various imaging modalities to facilitate ECVS-guided CNA when conventional femoral retrograde access to the IJVs is not feasible (Figure 4).

**Figure 5:**
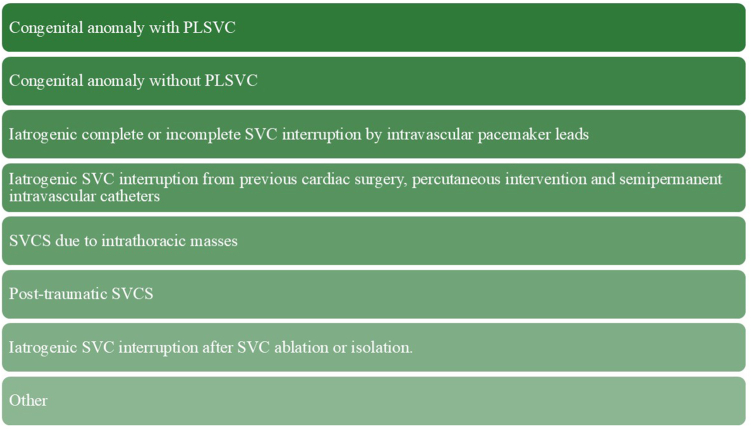
Possible causes of superior vena cava and brachiocephalic vein interruption during right- and left-sided attempts at extracardiac vagal nerve stimulation–guided cardioneuroablation. PLSVC = persistent left superior vena cava; SVC = superior vena cava; SVCS = superior vena cava syndrome.

The SVC and the left and right brachiocephalic veins may be affected by a wide range of congenital and acquired abnormalities (Figure 5). Congenital anomalies involving the brachiocephalic veins, their connections to the SVC, and their drainage into the cardiac chambers have important clinical implications, particularly in the field of cardiovascular interventional electrophysiology. These anomalies may be asymptomatic and occur in isolation, but they can also present with clinical symptoms and can be associated with other congenital heart defects and abnormal central venous connections.^5^ Therefore, in the absence of advanced preprocedural imaging, such as computed tomography or magnetic resonance imaging, recognition of these cardiovascular anomalies may be challenging. PLSVC is the most common systemic venous anomaly, affecting approximately 0.2%–2.0% of the general population.^6^^,^^7^ It may complicate procedures such as pacemaker implantation, TLE, and catheter ablation, but it does not preclude their successful completion.^8^ This case series confirms that retrograde access to the IJV via PLSVC is both feasible and effective in patients undergoing ECVS-guided CNA.

Disturbances in venous drainage to the RA may arise not only from congenital anomalies but also from acquired conditions that develop over time, leading to the manifestation of SVCS. Elevated upper-body venous pressure caused by impaired outflow to the SVC and venous congestion can manifest as swelling of the upper extremities, face, or neck, as well as dyspnea, cough, and dilated chest vein collaterals. SVCS may be caused by neoplastic changes or the presence of electrodes and semipermanent intravascular catheters.^9^^,^^10^ The incidence of lead-related moderate-to-severe stenosis or complete occlusion in patients undergoing TLE has been reported as 38.2% for the subclavian vein, 22.5% for the brachiocephalic vein, and 1.0% for the SVC.^11^ Severe SVCS is one of the major clinical manifestations of venous interruption; therefore, a multidisciplinary Heart Team approach is essential when planning such procedures. Although the primary goal remains to perform effective ECVS-guided CNA, symptomatic SVCS often necessitates adjunctive percutaneous interventions, including balloon venoplasty and stenting—typically preceded by TLE.^12^^,^^13^ In some cases, complete removal of the cardiac stimulation system may also be required.^14^ Final reassessment of the indication for permanent pacemaker therapy can be conducted unilaterally, even before TLE and restoration of venous continuity between the SVC, brachiocephalic veins, and IJVs. Although pacemaker implantation remains the gold standard for the treatment of patients with sick sinus syndrome or AVB, ongoing research is evaluating the potential advantages of CNA as an alternative to permanent pacing.^15^

Reproducibility of ECVS is essential for evaluating procedural performance and assessing the efficacy of CNA, particularly in high-risk patients requiring control procedures. However, postprocedural ECVS has several limitations, including the absence of additional electrophysiological or clinical signs of vagal nerve capture. Moreover, bilateral IJV cannulation before and after CNA was achieved in only 80% of cases, with a significantly higher rate of successful unilateral cannulation on the right side than the left.^16^^,^^17^

One of the indirect methods for confirming ECVS capture is the reproducible induction of tearing from the ipsilateral eye, accompanied by a nonsignificant reduction in sinus rhythm, prolongation of the PQ interval, or the appearance of Mobitz I AVB near the stable Wenckebach point.^18^ Another indirect method for confirming pacing with a reproducible site of the vagal nerve course in the cervical region involves ultrasound-guided cannulation of the IJV, using consistent anatomic landmarks and direct visualization of the catheter tip in close proximity to the vagal nerve. However, a key limitation of this approach is the difficulty in achieving timely and successful cannulation of the left IJV, which complicates accurate catheter navigation. In patients undergoing control ECVS or in high-risk cases, anatomic anomalies, thrombosis, or stenosis of the IJV should be excluded.

**Figure 6:**
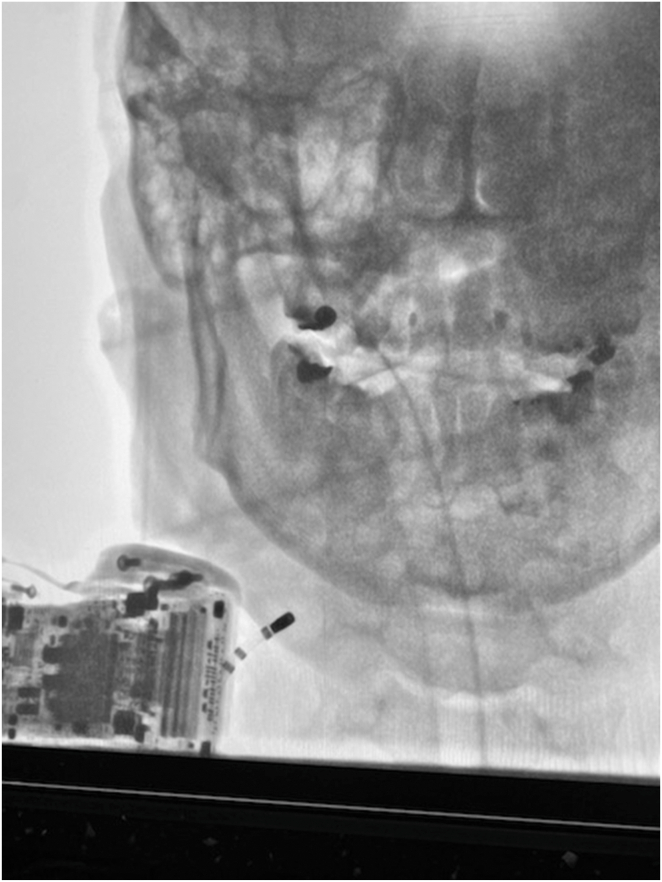
Ultrasound assessment of vagus nerve position in relation to the course of the internal jugular vein.

**Figure 7:**
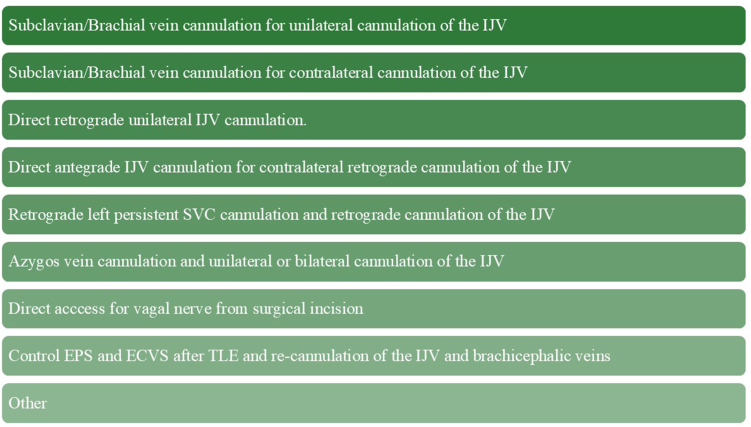
Alternative vascular access sites and maneuvers for internal jugular vein cannulation during extracardiac vagal nerve stimulation–guided cardioneuroablation in cases of superior vena cava and/or brachiocephalic vein interruption. ECVS = extracardiac vagal nerve stimulation; EPS = electrophysiology study; IJV = internal jugular vein; SVC = superior vena cava; TLE = transvenous lead extraction.

The recommended technique for ECVS involves introducing a pacing electrode under fluoroscopic guidance into the right and left IJVs, advancing it to the level of the jugular foramen (Figure 6). However, this approach has some limitations, including the inability to visualize the vagus nerve and the requirement for continuous fluoroscopy during electrode positioning.^19^^,^^20^ The lack of vagus nerve visualization may lead to inconclusive results, particularly if no vagal response is observed before CNA. In such cases, it may be unclear whether the absence of response is caused by intrinsically low vagal tone or, more likely, a failure to capture the vagus nerve owing to anatomic variability. These limitations may be overcome with ultrasound-guided ECVS, as demonstrated by Wileczek et al^21^ and Piotrowski et al,^22^ who confirmed that this technique is feasible and achieves full (binodal) vagal responses significantly more often than fluoroscopy-guided ECVS. ECVS-guided CNA has shown high periprocedural efficacy, with a success rate of 96.2%, and approximately 77% of patients remain symptom-free at 1-year follow-up (ie, no recurrence of syncope or second-degree AVB or higher).^23^ In some cases, alternative access sites may be necessary to perform ECVS (Figure 7).

## Conclusion

CNA is an effective therapeutic option for the treatment of VMB.^24^^,^^25^ Advances in diagnostic imaging and procedural techniques now allow CNA to be performed safely, even in patients with interrupted SVC drainage, regardless of its etiology. Although the prevalence of congenital anomalies involving the SVC may remain relatively constant, flow disturbances and obstructions within this part of the venous system are increasingly common. One of the primary causes is the presence of transvenous pacemaker leads, which are frequently implanted in patients with hypervagotonia. This raises important clinical questions about the long-term necessity of maintaining such pacing systems in patients who may be candidates for CNA. As novel therapeutic options such as CNA continue to evolve, new challenges arise that need to be addressed.

## Disclosures

The authors have no conflicts of interest to disclose.
